# Ceftriaxone Preserves Glutamate Transporters and Prevents Intermittent Hypoxia-Induced Vulnerability to Brain Excitotoxic Injury

**DOI:** 10.1371/journal.pone.0100230

**Published:** 2014-07-11

**Authors:** Rekha Jagadapillai, Nicholas M. Mellen, Leroy R. Sachleben, Evelyne Gozal

**Affiliations:** 1 Department of Pediatrics – KCHRI, University of Louisville, Louisville, Kentucky, United States of America; 2 Department of Pharmacology and Toxicology, University of Louisville, Louisville, Kentucky, United States of America; 3 Department of Physiology and Biophysics, University of Louisville, Louisville, Kentucky, United States of America; University G. D'Annunzio, Italy

## Abstract

Hypoxia alters cellular metabolism and although the effects of sustained hypoxia (SH) have been extensively studied, less is known about chronic intermittent hypoxia (IH), commonly associated with cardiovascular morbidity and stroke. We hypothesize that impaired glutamate homeostasis after chronic IH may underlie vulnerability to stroke-induced excitotoxicity. P16 organotypic hippocampal slices, cultured for 7 days were exposed for 7 days to IH (alternating 2 min 5% O_2_ - 15 min 21% O_2_), SH (5% O_2_) or RA (21% O_2_), then 3 glutamate challenges. The first and last exposures were intended as a metabolic stimulus (200 µM glutamate, 15 min); the second emulated excitotoxicity (10 mM glutamate, 10 min). GFAP, MAP2, and EAAT1, EAAT2 glutamate transporters expression were assessed after exposure to each hypoxic protocol. Additionally, cell viability was determined at baseline and after each glutamate challenge, in presence or absence of ceftriaxone that increases glutamate transporter expression. GFAP and MAP2 decreased after 7 days IH and SH. Long-term IH but not SH decreased EAAT1 and EAAT2. Excitotoxic glutamate challenge decreased cell viability and the following 200 µM exposure further increased cell death, particularly in IH-exposed slices. Ceftriaxone prevented glutamate transporter decrease and improved cell viability after IH and excitotoxicity. We conclude that IH is more detrimental to cell survival and glutamate homeostasis than SH. These findings suggest that impaired regulation of extracellular glutamate levels is implicated in the increased brain susceptibility to excitotoxic insult after long-term IH.

## Introduction

Restricted oxygen delivery alters brain cellular metabolism and increases astrocyte glucose uptake and lactate release to maintain viable energy levels and neuronal survival [1], [2]. Although the effects of sustained hypoxia (SH) have been extensively studied, less is known about chronic intermittent hypoxia (IH) that has been shown to increase cardiovascular risks, and is commonly seen in diseases such as obstructive sleep apnea (OSA).

OSA has been identified as an important risk factor for stroke, independent of other risks factors such as hypertension, increasing the outcome severity and functional consequences [3], [4], [5], [6]. Increased susceptibility to stroke in OSA patients has been mainly attributed to hypoxia-induced hemodynamic changes, as obstructive respiratory events elicit sympathetic and parasympathetic activation [6], [7], [8], that can be reversed by continuous positive airway pressure (CPAP) [9]. However, CPAP has not been unequivocally shown to decrease stroke susceptibility or improve stroke outcome [6]. Therefore additional cellular mechanisms may be induced by IH and play a role in OSA patients' vulnerability to ischemic injury.

The role of excitotoxicity in the pathogenesis of ischemic brain disease has been widely reported [10] and elevated glutamate levels have been found in plasma and in cerebrospinal fluid of stroke patients [11]. Acute ischemia, as occurs during stroke, induces the release of glutamate and down-regulates glutamate transporter expression, critical to the regulation of intrasynaptic glutamate [11], [12], [13]. However, the effect of long-term milder sustained or intermittent hypoxia on these transporters is unknown.

Astrocytes maintain extracellular potassium/glutamate homeostasis and synaptic function during stroke [14], [15]. In addition, astrocytes are critical for recovery after stroke and excitotoxicity and supply energy substrates for synaptic recovery following hypoxic injury [14], [16]. Glutamate transporters, mainly GLAST (EAAT1) and GLT1 (EAAT2), play a critical role in the process [17], [18]. EAAT2, primarily expressed in astrocytes, has been shown to be responsible for up to 90% of all glutamate uptake activity in the brain [19]. EAAT3, expressed mainly in neurons at very low levels, has been shown to play a role in neuronal metabolism rather than in glutamate transport [20]. The β-lactam antibiotic, ceftriaxone, increases GLT1 expression and function in rodents as well as in primary human fetal astrocytes and is neuroprotective *in vivo* in models of acute ischemia [21], [22]. Therefore, increasing glutamate transporter expression during chronic exposure to hypoxia may decrease brain susceptibility to hypoxia and to excitotoxic injury.

These processes are difficult to study in commonly used preparations: cell culture models exclude cellular interactions, while animal models do not allow identification and modulation of cellular and molecular mechanisms. Incomplete ischemia is a common consequence of cerebral artery occlusion and glutamate uptake is critical to neuronal recovery after ischemia thus is a limiting factor in glutamate toxicity [14]. To determine whether glutamate excitotoxicity and glutamate transport play a role in OSA patient's vulnerability to stroke, we exposed organotypic slices [23],[24] to long term IH and SH. To evaluate the effect of IH and SH on slices survival to excitotoxicity, we determined slices baseline response to a low glutamate challenge after 7 days exposure to SH and IH, then to a high glutamate concentration, as may occur in a stroke. To assess whether IH or SH exposure affected slices' ability to recover from excitotoxic challenge, high glutamate was washed away and a low glutamate concentration, similar to the first challenge, was applied. Our findings suggest that long term exposure to IH but not to SH alter glial glutamate transporter expression and compromises cell viability and brain response to excitotoxic insult that is critical during stroke. The beneficial effect of ceftriaxone treatment indicates that impaired glutamate transport plays a critical role in IH-induced vulnerability to ischemic brain injury.

## Materials and Methods

### Ethics Statement

Organotypic hippocampal slice cultures were prepared from 16-day-old Sprague-Dawley rats (Charles Rivers) as described in Stoppini et al., 1991 [23]. Rats were anaesthetized by intraperitoneal Nembutal injection, decapitated as approved by the University of Louisville Institutional Animal Use and Care Committee (protocol # 10100), and in agreement with the NIH guide for the care and use of laboratory animals.

### Organotypic slice culture and experimental protocol

400 µm dorsal hippocampal slices were cut in ice cold dissection medium (HBSS with 25 mM HEPES and 6% glucose), using a McIlwain tissue chopper. Slices were cultured onto inserts (Millicell-CM, Millipore, Billerica MA), with growth media (50% MEM (Invitrogen, Carlsbad, CA), 25% horse serum, 25% HBSS, 20 mM HEPES, 1 mM glutamine, and 5 mg/ml glucose) in a humidified incubator with 5% CO2 at 37 0C. Cultures were fed twice a week and grown for 7 days in room air (21% O2; RA) to lower microglial activation. Slices treated with 100 µM Ceftriaxone (Sigma) or with vehicle were exposed for 7 additional days to RA, mild sustained (SH; 5% O2, 5% CO2, balanced N2), or intermittent (IH; alternating 20 min 5% O2,- 10 min RA, 5% CO2, balanced N2) hypoxia, using a custom designed computer controlled incubator chamber (Biospherix, Redfield, NY), as previously performed in our laboratory [25]. Slices were collected immediately after exposure for histological study, or incubated with propidium iodide (PI). Baseline images were captured after 30 min PI incubation to assess slices viability prior to treatment with 3 short bouts of glutamate exposure, with a 20 min time-interval for capturing the images.

### Cell viability

Viability was assessed using:

1) Propidium iodine uptake (red) was used to show cell death in slices exposed to RA, IH and SH as well as to evaluate the effect of the various glutamate challenges on cell viability. Propidium iodide (PI; 5 µM; 30 min incubation) was added to the slices at the end of the hypoxic exposure to assess baseline viability, then after each experimental glutamate treatment. Total number of viable cells was shown by Fluorescein diacetate hydrolysis staining (FDA; 10 µg/ml; 30 min incubation; green). Images were captured using a laser scanning confocal microscope (Leica-TCS SL, Germany) and each image was averaged four times. Images were numbered so the person performing the analysis was blinded and unaware of the various experimental conditions. PI- stained cells were counted using custom machine-vision software that detected labeled cells using waterfall thresholding. At least 4 different fields/slice were counted and averaged for each experimental condition.

2) LDH release into the media was measured to assess cell death in slices, using LDH Cytotoxicity Detection Kit (Clontech, Mountain View, CA). Media were collected at the end of exposure and stored at -20°C. Samples were diluted 1∶10 and LDH release was quantified according to manufacturer's instructions. Absorbance (O.D.492 nm, reference wavelength 690 nm) was measured using a multiwall plate reader (Multiskan EX, Thermo Fisher Scientific, Vantaa, Finland).

### Immunofluorescence

Slices exposed to RA, IH or SH were fixed in 4% PFA and incubated overnight in primary antibody at 4°C (EAAT1 and EAAT2 (Abcam, Cambridge, MA; 1∶100 and 20 µg/ml); Map2 (Sigma, St.Louis, MO; 1∶100); GFAP (Cell signaling, Danver MA; 1∶200), washed with PBSTx, then incubated overnight with Cy3/FITC–tagged anti-rabbit/mouse secondary antibody, at room temperature in the dark. All slices were counterstained with DAPI to evaluate cellular density. Slices were washed with PBSTx, and visualized using a laser scanning confocal microscope (Leica-TCS SL, Germany). Fluorescent intensity was analyzed by a person blinded to the various experimental conditions, using of Adobe Photoshop for quantification, as previously described [29], and expressed as intensity per unit area.

### Western Blotting

Slices were homogenize in lysis buffer (0.4% NP-40, 1% glycerol, 50 mM tris at pH 7.5, 1 mM Na orthovanadate, 150 mM NaCl, 10 mM EDTA, 100 mM NaF, 20 mg/mL leupeptin, 10 mg/mL aprotinin, 0.5 mM PMSF). Protein concentrations were measured using the BioRad DC Protein Assay Kit (BioRad, Temecula, CA). Equal amounts of proteins were separated on 10% tris-glycine gels (Invitrogen, Carlsbad, CA) and transferred onto nitrocellulose membranes for immunodetection with antibodies against EAAT1 (Cell Signaling, Danver, MA), and EAAT2 (Abcam, Cambridge, MA). Proteins were visualized with SuperSignal West Pico chemiluminescence (Thermo Scientific Pierce, Rockford, IL). Equal loading was verified by reprobing the membranes with β-actin antibody. Equal transfer of proteins onto nitrocellulose membranes was verified by Ponceau-S staining. Immunoblots of slices lysates were repeated using slices obtained from at least three separate experiments.

### Statistics

SPSS IBM, v19 statistical software was used for data analysis. For viability studies, cell count data averaged over all experiments, averaging 4 fields per slice were analyzed using One-way ANOVA, and compared at baseline, at first 200 µM, at 10 mM and at second 200 µM glutamate exposure. Differences among the various glutamate concentrations were compared within each of the three hypoxia conditions for Ceftriaxone-treated or untreated slices, using One-way ANOVA, followed by Tukey HSD *post hoc* tests. Immunofluorescence quantification was analyzed using one-way ANOVA followed by Tukey HSD multiple comparison tests.

## Results

### 1. Effect of hypoxia on cell viability and susceptibility to glutamate

**Figure 1:**
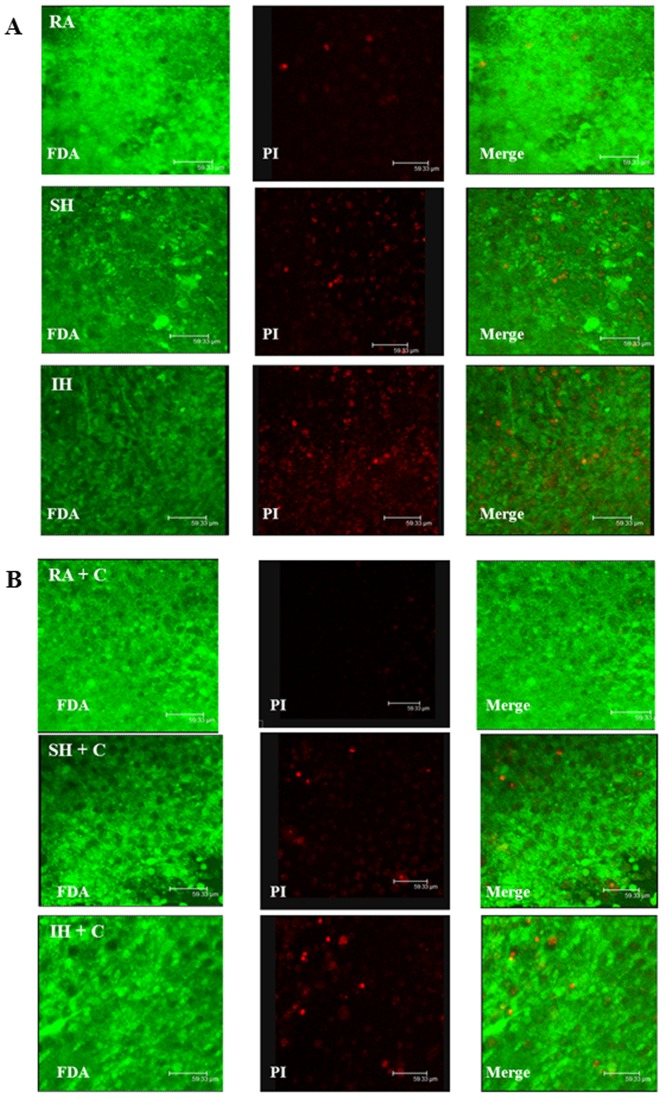
Long term sustained or Intermittent hypoxia decreases cell viability. Propidium iodide (red) and Fluorescein diacetate (green) staining of slices exposed to 7 days RA, SH or IH (A) without and (B) with 100 µM ceftriaxone (n = 4-6).

**Figure 2:**
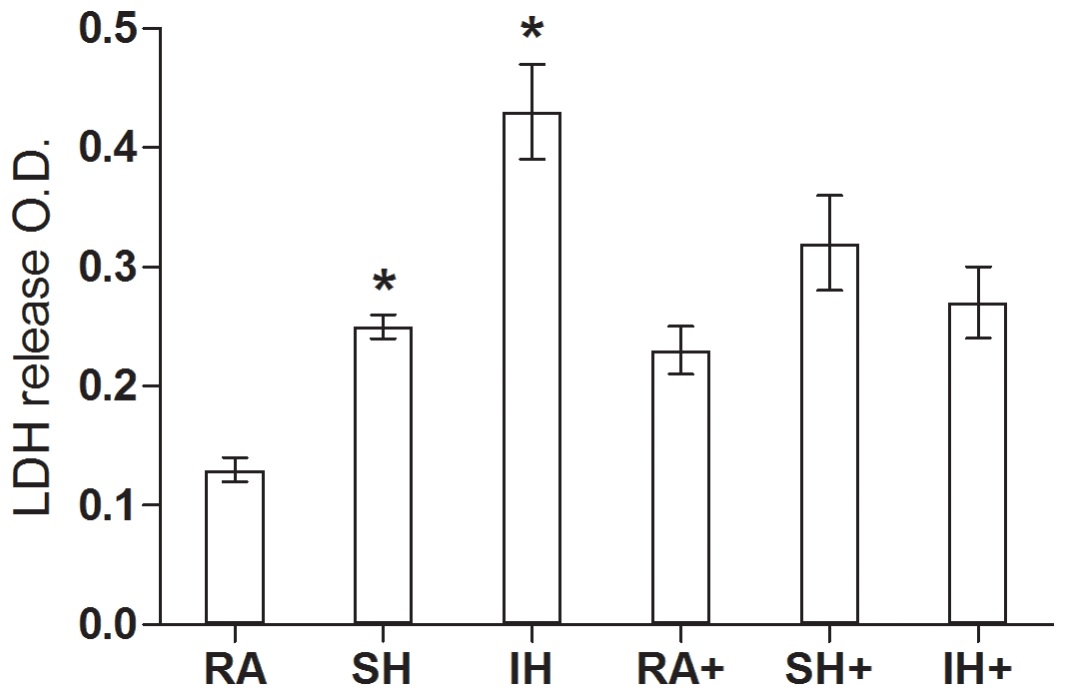
Long term sustained or Intermittent hypoxia decreases cell viability. LDH release in the supernatants of slices exposed to 7 days RA, SH or IH with (+) or without 100 µM ceftriaxone, presented as mean + SEM (n = 5–9). * RA<SH (p = 0.01). * RA<IH (p = 0.02).

**Figure 3:**
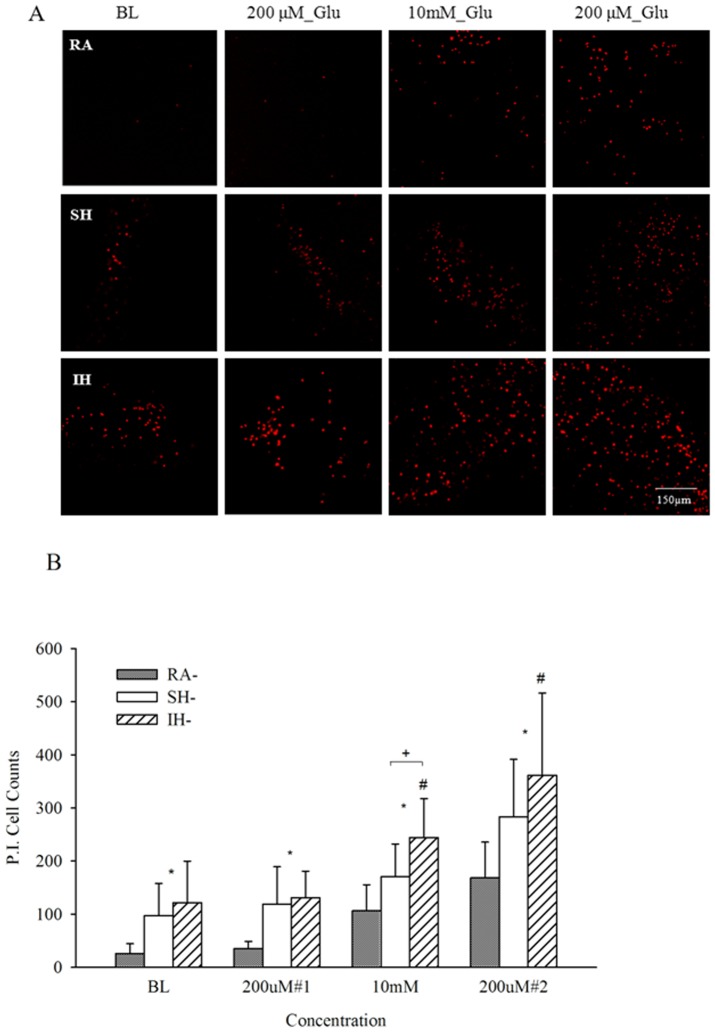
Intermittent hypoxia decreases cell viability and impairs glutamate response. Propidium iodide staining (A) and quantification of PI positive cells presented as mean + SD (B) of slices exposed to 7 days RA, SH or IH at baseline (BL), after 200 µM glutamate, and 10 mM glutamate, followed by a second 200 µM glutamate challenge. n = 12–18. *: At all concentrations RA^-^ < SH^-^ & IH^-^ (p<.001). ^+^ At 10mM: SH^-^ < IH^-^ (p<.05). ^#^ At 10 mM & 200 µM#2: BL < SH^-^ & IH^-^ (p<.05 and p<.001 respectively).

Cell viability was assessed, to determine the effect of long term exposure to IH and SH compared to RA. While in a cell culture model it would be relatively simple to quantify the total number of cells, cell density and slice architecture does not allow exact quantification in our organotypic slice model. Therefore, we have counterstained PI stainings after the various hypoxic exposures, using green hydrolyzed FDA staining. Living cells actively convert the non-fluorescent FDA into the green fluorescent fluorescein. Figure 1A shows decreased green fluorescence intensity after SH exposure, further decreasing after IH. In contrast, PI staining that stains damaged cells nuclei in red, increased in SH further increasing in IH. Confirming the PI data shown in Figure 1A, LDH release showed similar changes in viability, significantly increasing after SH and IH (Figure 2). To assess the effect of long term IH and SH on slice response to excitotoxicity, we determined slices baseline response to a non-excitotoxic glutamate challenge after 7 days exposure to RA, SH and IH, then to a high glutamate (10 mM) concentration, as may occur in a stroke. To assess whether slices exposed to IH or SH can recover from excitotoxic challenge, a low glutamate concentration, similar to the first challenge was then applied. Based on previous experimental evidence, the first and last glutamate exposures (200 µM glutamate for 15 min) should elicit a metabolic increase rather than excitotoxicity, as previously described in cultured astrocytes [26], [27]; the second glutamate exposure (10 mM glutamate for 10 min) emulates excitotoxic insult, as 10-20 mM glutamate have previously been shown in our laboratory to induce excitotoxicity in slices [28], hence 10 mM was selected as a concentration that elicited moderate but not extensive excitotoxic cell death, as could occur in a transient ischemic event. Cell death assessed by PI was significantly higher at baseline, confirming the results showed in Figure 1A, and after each glutamate challenge in slices exposed to IH or SH, compared to RA (Figure 3A, 3B). As expected, the first 200 µM glutamate challenge that was intended as a metabolic stimulus [26], [27], did not significantly affect viability in any of the conditions compared to their respective baseline. In contrast, excitotoxicity (10 mM glutamate) significantly increased cell death in all three conditions, with significantly higher toxicity in IH. Finally, the last 200 µM glutamate challenge significantly enhanced cell death in all three experimental conditions relative to baseline, and while the trend of higher IH toxicity was still apparent, there were no significant differences between conditions at this stage (Figure 3A, 3B).

### 2. Effect of ceftriaxone on cell viability and susceptibility to glutamate

Ceftriaxone has been shown to be neuroprotective in various models of ischemia, by upregulating glutamate transporters expression and activity. Therefore, we examined whether ceftriaxone, administered all along the hypoxic exposures, could prevent SH- and IH-induced cell death and improve tolerance to glutamate exposures.

**Figure 4:**
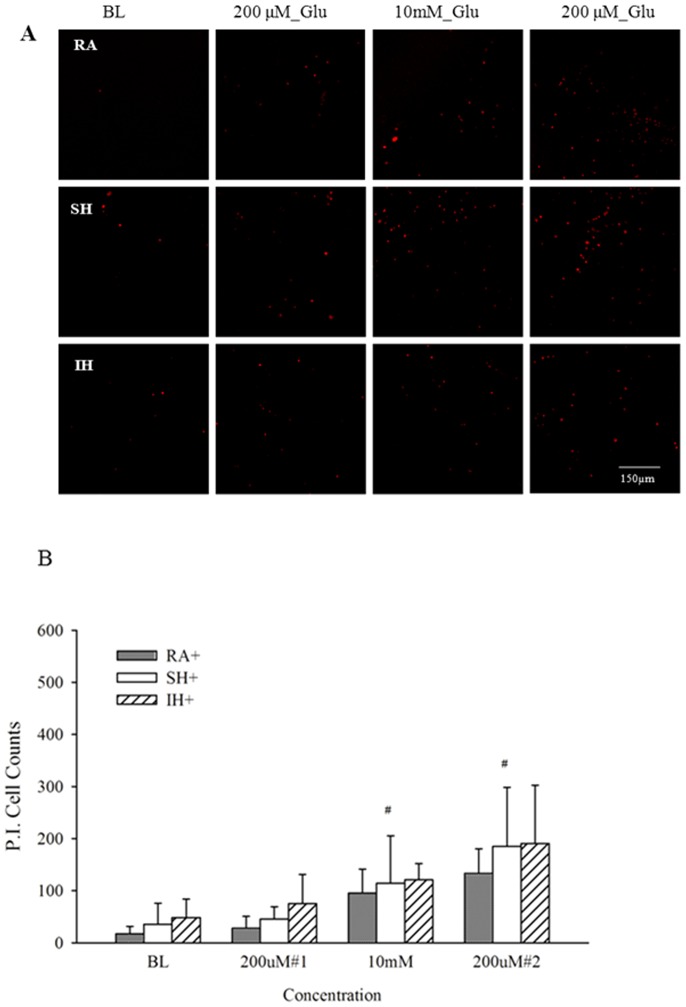
Ceftriaxone prevents hypoxia-induced cell death and improves tolerance to excitotoxicity. Propidium iodide staining (A) and quantification of PI positive cells presented as mean + SD (B) of slices exposed to 100 µM ceftriaxone during 7 days RA, SH or IH at baseline (BL), after 200 µM glutamate, and 10 mM glutamate, followed by a second 200 µM glutamate challenge. baseline (BL) n = 9–12, ^#^ At 10 mM & 200 µM#2: RA^+^; IH^+^; SH^+^> their respective BL (p<.01).

**Figure 5:**
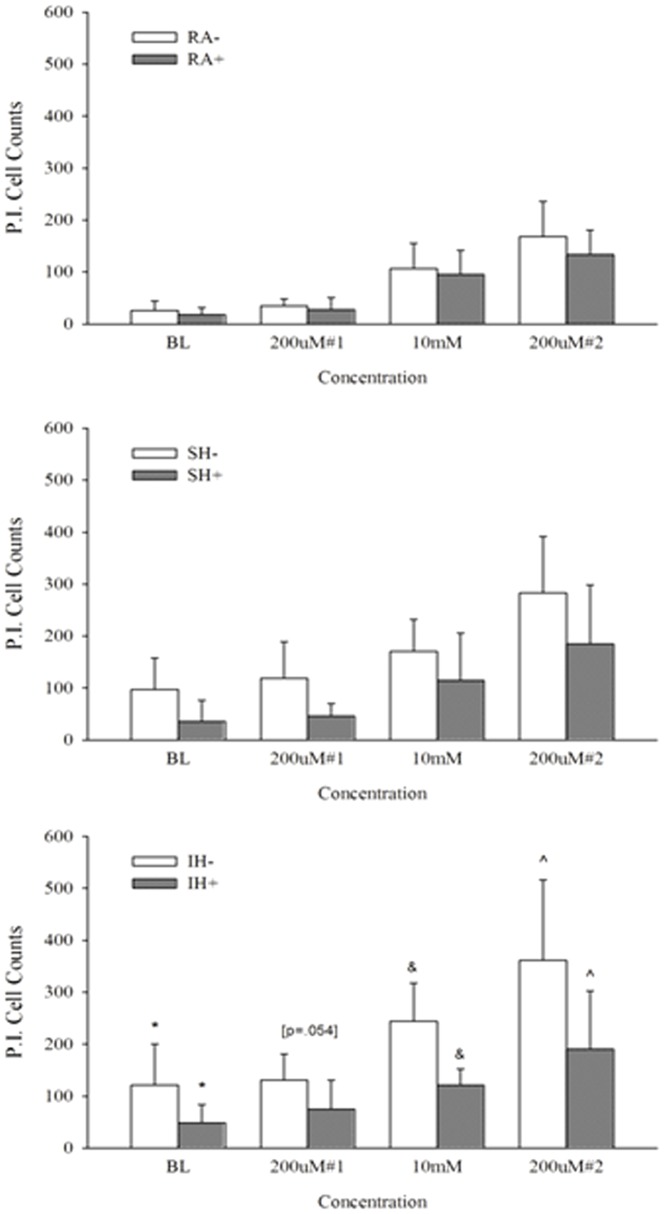
Ceftriaxone effect on cell tolerance to glutamate is significantly greater in IH-exposed slices. Propidium iodide staining quantification of ceftriaxone (+) treated slices exposed to RA, SH and IH at baseline (BL), after 200 µM glutamate, and 10 mM glutamate, followed by a second 200 µM glutamate challenge. n = 12–18 for RA^-^, SH^-^, IH^-^ and n = 9-12 for RA+, SH^+^, IH^+^; IH^-^ > IH^+^: *At BL (p = .005), ^&^At 10 mM (p<.001) & ∧At 200 µM#2 (p = .005).

FDA staining showed no significant difference in total number of cells in RA, IH and SH slices that were treated with ceftriaxone during their exposure (Figure 1B). However, PI-positive cells still increased after IH, compared to RA or SH albeit to a significantly lesser extent than in slices that were not treated with ceftriaxone Figure 1B. These data agreed with our LDH release results showing no significant difference in viability of slices after RA, IH or SH when slices were treated with ceftriaxone (Figure 2). Ceftriaxone prevented the increase in cell death at baseline and after each glutamate challenge in IH and SH slices, compared to their respective RA controls. Ceftriaxone treatment abrogated differences in viability between RA, IH and SH within each glutamate concentration (Figure 4). Similarly to ceftriaxone-untreated slices, the first 200 µM challenge did not increase cell death in any of the conditions. However, cytotoxicity at 10 mM and after the second 200 µM challenge, while greatly attenuated by ceftriaxone, remained significantly higher compared to their respective baseline in all three experimental conditions (Fig. 4B). These data suggest that ceftriaxone increased tolerance to glutamate and abrogated the difference in excitotoxic cell death between IH and SH-treated slices, but may not suffice to completely prevent excitotoxic insult. Graphic comparison of ceftriaxone-treated and untreated slices at each glutamate concentration, revealed no significant differences in the RA or SH-exposed slices, although a clear trend of decreased excitotoxicity was apparent in ceftriaxone-treated SH slices (Fig. 5). In contrast, ceftriaxone-treated IH slices had significantly lower cell death even after 10 mM glutamate and throughout the second 200 µM glutamate challenge (Fig. 5). These data suggest that impaired glutamate transport and uptake may play an important role in IH-induced vulnerability to excitotoxic injury.

### 3. Effect of hypoxia on glutamate transporters expression

**Figure 6:**
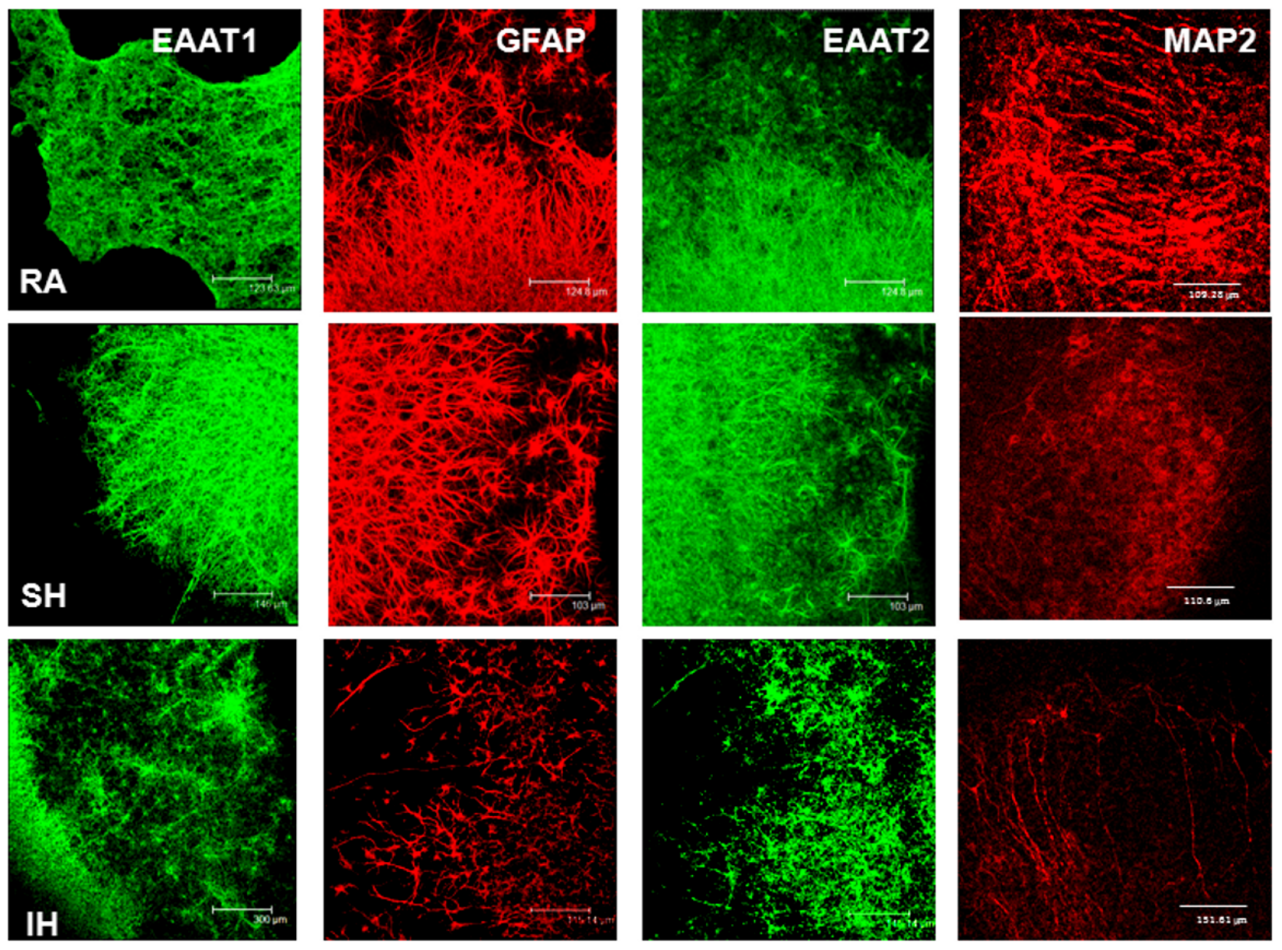
IH significantly decreases glutamate transporters, MAP2 and GFAP immunoreactivity. EAAT1, EAAT2, GFAP and MAP2 immunoreactivity in slices exposed to 7 days RA, SH or IH. EAAT1 and EAAT2 expression was unchanged by SH while significantly reduced in IH. MAP2 and GFAP expression decreased in both SH and IH.

**Figure 7:**
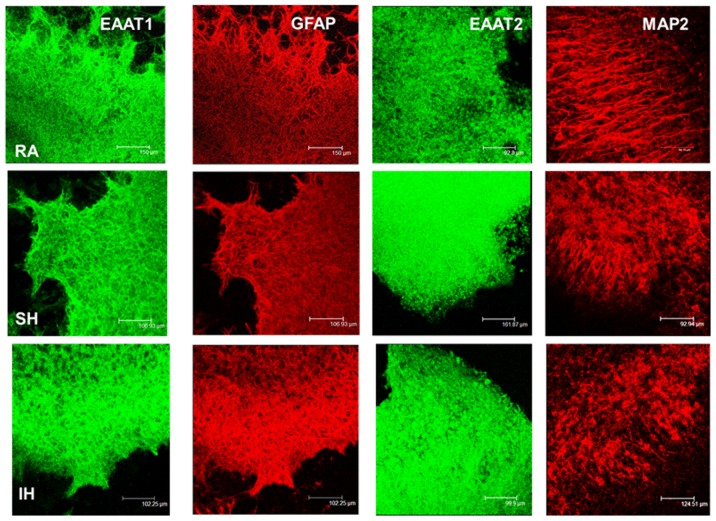
Ceftriaxone prevents IH-induced decrease in glutamate transporters, MAP2 and GFAP immunoreactivity. EAAT1, EAAT2, GFAP and MAP2 immunoreactivity in slices exposed to 7 days RA, SH or IH in presence of 100 µM ceftriaxone. Ceftriaxone treatment prevented the decrease in EAAT1, EAAT2 in IH, and of MAP2 and GFAP in both IH and SH.

**Figure 8:**
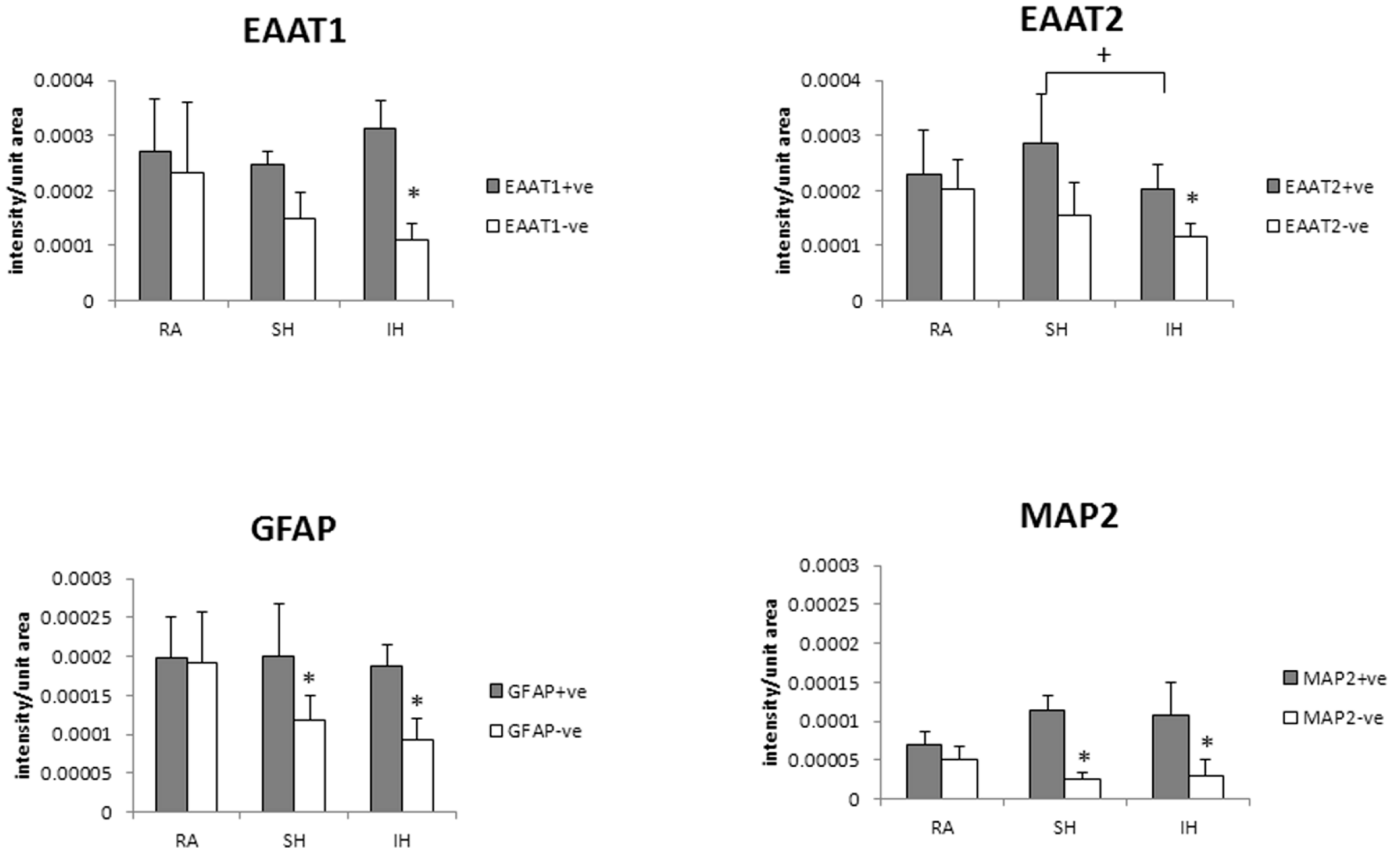
Graphic representation of glutamate transporters, MAP2 and GFAP immunoreactivity with or without ceftriaxone. Quantification of EAAT1, EAAT2, GFAP and MAP2 immunofluorescence per unit area of slices exposed to 7 days RA, SH or IH in presence (+ve) or in absence (-ve) of 100 µM ceftriaxone. Data are presented as mean immunofluorescence + SD. n =  4-13 for RA^+^, IH^+^, SH^+^; n = 5–11 for RA^-^, IH^-^, SH *: IH^-^ or SH^-^ <RA^-^ (p≤0.01). ^+^: IH^+^ < SH^+^ (p≤0.01).

**Figure 9:**
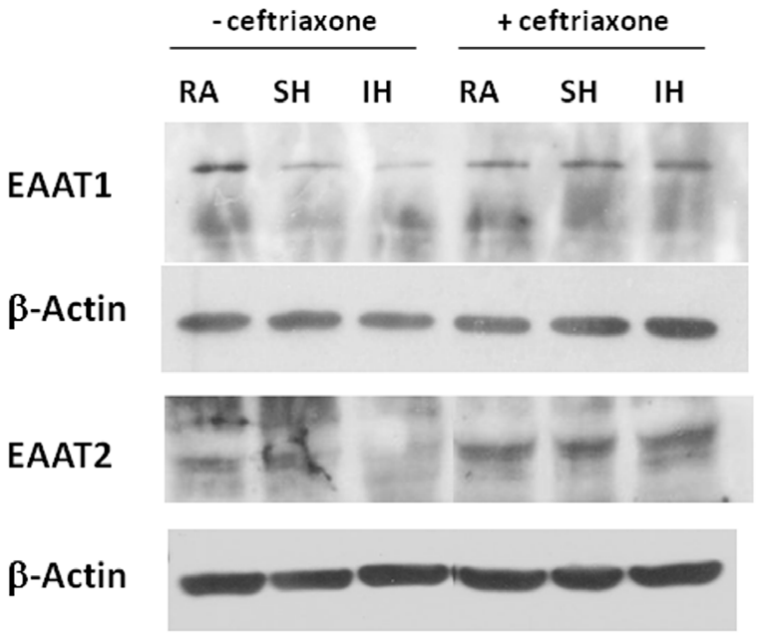
EAAT1 and EAAT2 immunoblotting of slices exposed to RA, SH and IH with or without ceftriaxone shows that ceftriaxone prevents IH-induced decrease in glutamate transporters. EAAT1 and EAAT2, immunoblotting of lysates from slices exposed to 7 days RA, SH or IH in absence or in presence of 100 µM ceftriaxone. Glutamate transporters expression decreased in IH-exposed slices. Ceftriaxone treatment increased EAAT1 and EAAT2 expression in all conditions and prevented EAAT1 and EAAT2 decrease in IH.

Our results showed that ceftriaxone reduced SH- and IH-exposed tissue lability to excitotoxicity. Therefore, to determine whether changes in EAAT1 and EAAT2 expression underlie the differences in cell viability in the various conditions, we assessed and quantified their expression in slices exposed to 7 days RA, IH or SH. Additionally, to determine whether long term SH and IH differentially affect neuronal or glial cells, MAP2 and GFAP immunoreactivity were assessed and quantified. Immunohistochemical stainings showed that EAAT1 and EAAT2 expression significantly decreased after IH and while there was a trend of decrease after SH, it did not reach statistical significance (Fig. 6). Therefore, IH appears to down-regulate glutamate transporters expression to a greater extent than SH exposure. Ceftriaxone treatment prevented EAAT1 and EAAT2 decrease (Fig. 7). Quantification of EAAT1 and EAAT2 immunoreactivity confirmed these results (Fig. 8). SH and IH exposure decreased GFAP and MAP2 expression compared to RA (Figures 6, 8), and ceftriaxone treatment prevented that decrease (Figures 7, 8). These results were confirmed by immunoblotting (Fig. 9). These data confirm our viability findings, showing that exposure to long term mild IH or SH significantly decreased cell viability at baseline (Figures 1, 3).

## Discussion

Our results show that long-term IH, even at a moderate level of hypoxia, decreases glutamate transporters expression, resulting in cytotoxicity and reduced tolerance to glutamate exposure. In contrast, long-term moderate SH, while affecting cell viability at baseline, only minimally affected glutamate transporter expression and cell viability after glutamate challenge. These results suggest that different mechanisms may be involved in IH- and SH-induced injury that may underlie higher vulnerability to excitotoxic insult in IH-exposed slices. Indeed, we had previously reported that IH and SH induce PC12 cell apoptosis via different mechanisms [25].

The use of organotypic slices in this study, preserves the interdigitated networks of neurons and astrocytes as well as soluble factors released from neurons that are essential for the expression of GLT1 [30], [31]. Brain slices are cultured at the air/media interface, directly exposed to the various gas profiles. This preparation provides adequate conditions for implementation of a physiologically relevant IH protocol allowing us to assess cellular changes that could contribute to increased vulnerability to stroke-induced excitotoxicity after exposure to various hypoxic profiles.

Preconditioning, using short bouts of ischemia or brief courses of IH, has been shown to protect against ischemic injury. IH protocols of short-term and moderate IH regimens have also been proposed in several studies as a promising therapeutic option to prevent and treat hypertension [32]. In contrast, repetitive severe and brief IH as occurs in OSA, have been shown to induce hypertension and cardiovascular disease [33]. In a recent study, Gong et al., reported IH-induced preconditioning, exposing rats to 6 h/day hypobaric hypoxia for 28 days and observed increased GLT1 expression that correlated with increased tolerance to ischemia [34]. However the IH protocol used in our study with constant repetitive IH during 7 days, rather mimics IH deleterious effects and induces a significant decrease in glutamate transporter expression. Therefore, different IH paradigms can produce remarkably divergent effects depending greatly on duration and intensity of hypoxia exposure, the number of hypoxia-reoxygenation bouts per day and the total days of the protocol.

GLT1 and GLAST are sodium-dependent glutamate transporters [35]. Decreased glutamate transporter expression has been reported after acute ischemic injury, resulting in elevated extracellular glutamate [12], [13]. During ischemia, the mitochondrial respiration chain is compromised, leading to energy failure that may reverse the transporter, carrying glutamate to the extracellular space and further contributing to excitotoxicity [36]. However, our hypoxic paradigm delivers a relatively mild hypoxic challenge (5% O_2_) that does not involve total O_2_ deprivation, thus moderately affects the mitochondrial respiration chain. Additionally, recurrent reoxygenation during IH preserves oxygen-dependent mitochondrial chain function preventing energy failure. Therefore, it is unlikely that reverse glutamate transport contributes to extracellular glutamate accumulation in our experimental conditions, and increased glutamate excitoxicity in IH-exposed slices is likely due to impaired transporter expression/activity rather than to transporter reversal.

Ceftriaxone, a well-known and well tolerated β-lactam antibiotic, has been reported to induce glutamate transporter expression in rat astrocytes cultures, in organotypic spinal cord slices and in vivo in rat brain and spinal cord [21], [22]. GLT1 upregulation contributes to preconditioning-induced neuroprotection against acute and delayed cell death resulting from brain ischemic injury in rats [37],[38]. In agreement with these studies, ceftriaxone prevented glutamate transporters expression decrease and significantly reduced excitotoxic cell death in all three conditions also abrogating the increased IH susceptibility to high glutamate challenge, when compared to SH-exposed slices. While most current therapies aim at improving stroke outcome, our data suggest that ceftriaxone preserves brain glutamate response during IH exposure and could potentially serve as a prophylactic therapy to improve brain tolerance to transient ischemic events as well as minimize stroke functional consequences in a population of patients at risk.

Studies using GLT1 null mice or antisense knockdown animals have shown that GLT1 is responsible for over 90% of glutamate clearance in the CNS [19]. Our findings show that expression of both glutamate transporters decreased in IH and to a lesser extent in SH and that excitotoxic injury is significantly greater in IH- than in SH-exposed slices. Ceftriaxone preserved both glial and neuronal cells, prevented the decreased EAAT1 and EAAT2 expression in IH and significantly reduced excitotoxic injury in IH-exposed slices, while moderately benefiting RA- or SH-exposed slices that showed no substantial decrease in glutamate transporter expression. These data suggest that while upregulation of the transporters is beneficial in all conditions, it is critical for IH-exposed slices while additional factors may underlie excitotoxic cell death in SH-exposed slices. Our data suggest that the intermittent characteristic of IH induces distinct signaling pathways leading to a decrease in EAAT2 expression and that ceftriaxone may not only alleviate excitotoxic damage in all three conditions, but also protects from IH-induced vulnerability. The mechanism underlying IH–induced glutamate transporters down-regulation remains to be elucidated.

Several potential mechanisms could interact to decrease the expression and function of glutamate transporters in IH. At the transcriptional level, abnormal mRNA splicing results in truncated EAAT2 mRNA splice products and dysfunctional proteins [39]. Additional post-translational modifications, such as redox modulation of EAAT2 reactive amino acids may impact the transporter function [40] and may occur in IH [41], [42]. Several transcription factors, such as NF-κB, upregulated during inflammation, have been shown to be regulated via redox modifications [43]. Experiments using 5′-deletion mutants of the EAAT2 promoter constructs identified one of the 4 NF-κB binding sites as a critical regulator of ceftriaxone-induced EAAT2 transcription in primary human fetal astrocytes [22], [44]. Cardiovascular and cerebrovascular inflammation involving NF-κB activation during OSA has been reviewed [45], [46] and has also recently been described in HeLa cells exposed to IH [47] and *in vivo* in C57BL6 mice exposed to 14 days IH [48]. However, consequences of IH-induced NF-κB changes in the brain and their effect on glutamate homeostasis have not been investigated.

Clinical trials with glutamate receptor antagonists that would be expected to prevent excitotoxicity have been associated with untoward side effects and little clinical benefit. Thus, therapies aiming at enhancing glutamate transporter expression and activity merit exploration. Ceftriaxone, upregulating transporter expression with minimal toxicity, could be administered orally to patients at risk as a preventive therapy or during the recovery phase after a stroke. Ceftriaxone is currently in clinical trials for stroke therapy and has been shown to be neuroprotective both *in vivo* and *in vitro* in various models of acute ischemic injury [21], [22], but has not been used as a prophylactic treatment in any pathological condition involving IH.

In summary, impaired glutamate transport has been implicated in multiple neurodegenerative diseases, prompting the development of novel therapeutics to increase transporters expression. However, our study presents a first report of decreased astroglial glutamate transporters as a result of a chronic, non-degenerative pathology, involving IH. Our findings suggests that preventive strategies to prevent the loss of glutamate transporters, could be implemented in such patients presenting cardiovascular alterations putting them at increased risk for stroke, and could improve their ability to withstand potential brain ischemic events.

## References

[1] Schurr A, Miller JJ, Payne RS, Rigor BM (1999) An increase in lactate output by brain tissue serves to meet the energy needs of glutamate-activated neurons. J Neurosci 19: 34–39. 9870935 10.1523/JNEUROSCI.19-01-00034.1999 PMC6782362

[2] Vega C, Sachleben R, Gozal D, Gozal E (2006) Differential metabolic adaptation to acute and long-term hypoxia in rat primary cortical astrocytes. J Neurochem 97: 872–883. 16573648 10.1111/j.1471-4159.2006.03790.x

[3] Yaggi HK, Concato J, Kernan WN, Lichtman JH, Brass LM, et al (2005) Obstructive sleep apnea as a risk factor for stroke and death. N Engl J Med 353: 2034–2041. 16282178 10.1056/NEJMoa043104

[4] Arzt M, Young T, Finn L, Skatrud JB, Bradley TD (2005) Association of sleep-disordered breathing and the occurrence of stroke. Am J Respir Crit Care Med 172: 1447–1451. 16141444 10.1164/rccm.200505-702OC PMC2718439

[5] Yan-fang S, Yu-ping W (2009) Sleep-disordered breathing: impact on functional outcome of ischemic stroke patients. Sleep Med 10: 717–719. 19168390 10.1016/j.sleep.2008.08.006

[6] Calvin AD, Somers VK (2009) Obstructive sleep apnea and risk of stroke: time for a trial. Nat Clin Pract Cardiovasc Med 6: 90–91. 19065125 10.1038/ncpcardio1418

[7] Somers VK, Dyken ME, Mark AL, Abboud FM (1992) Parasympathetic hyperresponsiveness and bradyarrhythmias during apnoea in hypertension. Clin Auton Res 2: 171–176. 1498563 10.1007/BF01818958

[8] Kato M, Adachi T, Koshino Y, Somers VK (2009) Obstructive sleep apnea and cardiovascular disease. Circ J 73: 1363–1370. 19564701 10.1253/circj.cj-09-0364

[9] Martinez-Garcia MA, Soler-Cataluna JJ, Ejarque-Martinez L, Soriano Y, Roman-Sanchez P, et al (2009) Continuous positive airway pressure treatment reduces mortality in patients with ischemic stroke and obstructive sleep apnea: a 5-year follow-up study. Am J Respir Crit Care Med 180: 36–41. 19406983 10.1164/rccm.200808-1341OC

[10] Dirnagl U, Iadecola C, Moskowitz MA (1999) Pathobiology of ischaemic stroke: an integrated view. Trends Neurosci 22: 391–397. 10441299 10.1016/s0166-2236(99)01401-0

[11] Aliprandi A, Longoni M, Stanzani L, Tremolizzo L, Vaccaro M, et al (2005) Increased plasma glutamate in stroke patients might be linked to altered platelet release and uptake. J Cereb Blood Flow Metab 25: 513–519. 15660099 10.1038/sj.jcbfm.9600039

[12] Rossi DJ, Oshima T, Attwell D (2000) Glutamate release in severe brain ischaemia is mainly by reversed uptake. Nature 403: 316–321. 10659851 10.1038/35002090

[13] Dallas M, Boycott HE, Atkinson L, Miller A, Boyle JP, et al (2007) Hypoxia suppresses glutamate transport in astrocytes. J Neurosci 27: 3946–3955. 17428968 10.1523/JNEUROSCI.5030-06.2007 PMC6672540

[14] Chen Y, Swanson RA (2003) Astrocytes and brain injury. J Cereb Blood Flow Metab 23: 137–149. 12571445 10.1097/01.WCB.0000044631.80210.3C

[15] Theodosis DT, Poulain DA, Oliet SH (2008) Activity-dependent structural and functional plasticity of astrocyte-neuron interactions. Physiol Rev 88: 983–1008. 18626065 10.1152/physrev.00036.2007

[16] Dronne MA, Grenier E, Dumont T, Hommel M, Boissel JP (2007) Role of astrocytes in grey matter during stroke: a modelling approach. Brain Res 1138: 231–242. 17274959 10.1016/j.brainres.2006.12.062

[17] Voutsinos-Porche B, Bonvento G, Tanaka K, Steiner P, Welker E, et al (2003) Glial glutamate transporters mediate a functional metabolic crosstalk between neurons and astrocytes in the mouse developing cortex. Neuron 37: 275–286. 12546822 10.1016/s0896-6273(02)01170-4

[18] Danbolt NC (2001) Glutamate uptake. Prog Neurobiol 65: 1–105. 11369436 10.1016/s0301-0082(00)00067-8

[19] Tanaka K, Watase K, Manabe T, Yamada K, Watanabe M, et al (1997) Epilepsy and exacerbation of brain injury in mice lacking the glutamate transporter GLT-1. Science 276: 1699–1702. 9180080 10.1126/science.276.5319.1699

[20] Holmseth S, Dehnes Y, Huand YH, Follin-Arbelet VV, Grutle NJ, et al (2012) The density of EAAC1 (EAAT3) glutamate transporters expressed by neurons in the mammalian CNS. J Neurosci 32: 6000–6013. 22539860 10.1523/JNEUROSCI.5347-11.2012 PMC4031369

[21] Rothstein JD, Patel S, Regan MR, Haenggeli C, Huang YH, et al (2005) Beta-lactam antibiotics offer neuroprotection by increasing glutamate transporter expression. Nature 433: 73–77. 15635412 10.1038/nature03180

[22] Lee SG, Su ZZ, Emdad L, Gupta P, Sarkar D, et al (2008) Mechanism of ceftriaxone induction of excitatory amino acid transporter-2 expression and glutamate uptake in primary human astrocytes. J Biol Chem 283: 13116–13123. 18326497 10.1074/jbc.M707697200 PMC2442320

[23] Stoppini L, Buchs PA, Muller D (1991) A simple method for organotypic cultures of nervous tissue. J Neurosci Methods 37: 173–182. 1715499 10.1016/0165-0270(91)90128-m

[24] Gahwiler BH, Capogna M, Debanne D, McKinney RA, Thompson SM (1997) Organotypic slice cultures: a technique has come of age. Trends Neurosci 20: 471–477. 9347615 10.1016/s0166-2236(97)01122-3

[25] Gozal E, Sachleben LR Jr, Rane MJ, Vega C, Gozal D (2005) Mild sustained and intermittent hypoxia induce apoptosis in PC-12 cells via different mechanisms. Am J Physiol Cell Physiol 288: C535–542. 15537711 10.1152/ajpcell.00270.2004

[26] Pellerin L, Magistretti PJ (1994) Glutamate uptake into astrocytes stimulates aerobic glycolysis: a mechanism coupling neuronal activity to glucose utilization. Proc Natl Acad Sci U S A 91: 10625–10629. 7938003 10.1073/pnas.91.22.10625 PMC45074

[27] Azarias G, Perreten H, Lengacher S, Poburko D, Demaurex N, et al (2011) Glutamte transport decreases mitochondrial pH and modulates oxidative metabolism in astrocytes. J Neurosci 31: 3550–3559. 21389211 10.1523/JNEUROSCI.4378-10.2011 PMC6622778

[28] Schurr A, Payne RS, Tseng MT, GozalE GozalD (2001) Excitotoxic preconditioning elicited by both glutamate and hypoxia and abolished by lactate transport inhibition in rat hippocampal slices. Neurosci Lett 307: 151–154. 11438386 10.1016/s0304-3940(01)01937-1

[29] Zheng S, Huang Y, Yang L, Chen T, Xu J, et al (2011) Uninephrectomy of diabetic OVE26 mice greatly accelerates albuminuria, fibrosis, inflammatory cell infiltration and changes in gene expression. Nephron Exp Nephrol 119: e21–32. 21659782 10.1159/000327586 PMC3701440

[30] Gegelashvili G, Civenni G, Racagni G, Danbolt NC, Schousboe I, et al (1996) Glutamate receptor agonists up-regulate glutamate transporter GLAST in astrocytes. Neuroreport 8: 261–265. 9051792 10.1097/00001756-199612200-00052

[31] Gegelashvili G, Danbolt NC, Schousboe A (1997) Neuronal soluble factors differentially regulate the expression of the GLT1 and GLAST glutamate transporters in cultured astroglia. J Neurochem 69: 2612–2615. 9375696 10.1046/j.1471-4159.1997.69062612.x

[32] Serebrovskava TV, Manukhina EB, Smith ML, Downey FH, Mallet RT (2008) Intermittent Hypoxia: Cause of or Therapy for Systemic Hypertension?. Exp Biol Med 233: 627–650. 10.3181/0710-MR-267 18408145

[33] Foster GE, Poulin MJ, Hanly PJ (2007) Intermittent hypoxia and vascular function: implications for obstructive sleep apnoea. Exp Physiol 92: 51–65. 17124276 10.1113/expphysiol.2006.035204

[34] Gong S-J, Chen L-Y, Zhang M, Gong J-X, Ma Y-X, et al (2011) Intermittent Hypobaric Hypoxia Preconditioning Induced Brain Ischemic Tolerance by Up-Regulating Glial Glutamate Transporter-1 in Rats. Neurochem Res 37: 527–537. 22076500 10.1007/s11064-011-0639-3

[35] Benarroch EE (2010) Glutamate transporters: diversity, function, and involvement in neurologic disease. Neurology 74: 259–264. 20083803 10.1212/WNL.0b013e3181cc89e3

[36] Grewer C, Gameiro A, Zhang Z, Tao Z, Braams S, et al (2008) Glutamate forward and reverse transport: from molecular mechanism to transporter-mediated release after ischemia. IUBMB Life 60: 609–619. 18543277 10.1002/iub.98 PMC2632779

[37] Zhang M, Li WB, Geng JX, Li QJ, Sun XC, et al (2007) The upregulation of glial glutamate transporter-1 participates in the induction of brain ischemic tolerance in rats. J Cereb Blood Flow Metab 27: 1352–1368. 17228332 10.1038/sj.jcbfm.9600441

[38] Liu YX, Zhang M, Liu LZ, Cui X, Hu YY, et al (2012) The role of glutamate transporter-1a in the induction of brain ischemic tolerance in rats. Glia 60: 112–124. 21971915 10.1002/glia.21252

[39] Lin CG, Bristol LA, Jin L, DykesHoberg M, Crawford T, et al (1998) Aberrant RNA processing in a neurodegenerative disease: the cause for absent EAAT2 a glutamate transporter in amyotrophic lateral sclerosis. Neuron 20: 589–602. 9539131 10.1016/s0896-6273(00)80997-6

[40] Trotti D, Rolfs A, Danbolt NC, Brown RH Jr, Hediger MA (1999) SOD1 mutants linked to amyotrophic lateral sclerosis selectively inactivate a glial glutamate transporter. Nat Neurosci 2: 427–433. 10321246 10.1038/8091

[41] Nisbet RE, Graves AS, Kleinhenz DJ, Rupnow HL, Reed AL, et al (2009) The role of NADPH oxidase in chronic intermittent hypoxia-induced pulmonary hypertension in mice. Am J Respir Cell Mol Biol 40: 601–609. 18952568 10.1165/rcmb.2008-0145OC PMC2677439

[42] Lavie L, Lavie P (2009) Molecular mechanisms of cardiovascular disease in OSAHS: the oxidative stress link. Eur Respir J 33: 1467–1484. 19483049 10.1183/09031936.00086608

[43] Haddad JJ (2002) Antioxidant and prooxidant mechanisms in the regulation of redox(y)-sensitive transcription factors. Cell Signal 14: 879–897. 12220615 10.1016/s0898-6568(02)00053-0

[44] Kim K, Lee SG, Kegelman TP, Su ZZ, Das SK, et al (2010) Role of excitatory amino acid transporter-2 (EAAT2) and glutamate in neurodegeneration: Opportunities for developing novel thearpeutics. J Cell Physiol 226: 2484–2493. 10.1002/jcp.22609 PMC3130100 21792905

[45] Lavie L (2005) Sleep-disordered breathing and cerebrovascular disease: a mechanistic approach. Neurol Clin 23: 1059–1075. 16243616 10.1016/j.ncl.2005.05.005

[46] Ryan S, Taylor CT, McNicholas WT (2009) Systemic inflammation: a key factor in the pathogenesis of cardiovascular complications in obstructive sleep apnoea syndrome? Postgrad Med J 85: 693–698. 20075410 10.1136/thx.2008.105577

[47] Ryan S, Taylor CT, McNicholas WT (2005) Selective activation of inflammatory pathways by intermittent hypoxia in obstructive sleep apnea syndrome. Circulation 112: 2660–2667. 16246965 10.1161/CIRCULATIONAHA.105.556746

[48] Arnaud C, Beguin PC, Lantuejoul S, Pepin JL, Guillermet C, et al (2011) The Inflammatory Pre-Atherosclerotic Remodeling Induced by Intermittent Hypoxia is Attenuated by RANTES/CCL5 Inhibition. Am J Respir Crit Care Med 184: 724–731. 21680945 10.1164/rccm.201012-2033OC PMC4901161

